# Vaccine responses to conserved regions of the HIV-1 proteome are associated with an increased capacity to inhibit multiple virus isolates ex vivo

**DOI:** 10.1186/1742-4690-9-S2-O64

**Published:** 2012-09-13

**Authors:** A Ashraf, J Kopycinski, H Cheeseman, F Lala, J Czyzewska-Khan, A Spentzou, DK Gill, M Keefer, J Excler, P Fast, P Hayes, JH Cox, J Gilmour

**Affiliations:** 1 International AIDS Vaccine Initiative (IAVI), Imperial College London, London, UK; 2 University of Rochester School of Medicine & Dentistry, Rochester, NY, USA; 3 International AIDS Vaccine Initiative (IAVI), New York, NY, USA

## Background

The majority of assays currently used to assess HIV-1 vaccine candidate immunogenicity in humans fail to predict protection against HIV-1 acquisition or control of viraemia. However, a correlation between in vivo and ex vivo control mediated by CD8+ T-cell populations has been described using an ex vivo virus inhibition assay (VIA) in chronically infected individuals and vaccinated non-human primates. Here we attempt to relate the specificity of vaccine-induced virus-specific CD8 responses to the inhibition of HIV-1 ex vivo.

## Methods

Using peptide epitope mapping, we assessed the breadth and specificity of CD8 T-cell responses induced by vaccination using two adenovirus serotype 35 (Ad35) vectors containing gag, reverse transcriptase, integrase and nef (Ad35-GRIN) and env (Ad35-ENV), respectively, derived from HIV-1 subtype A isolates. The conserved regions targeted by these 25 subjects were related to the capacity of vaccine-induced CD8 T-cells to inhibit replication of a cross-clade panel of HIV-1 isolates using the VIA.

## Results

A median of 4 peptides were recognised in vaccinated individuals (range 1-9). When related to the log reduction of p24 production as measured in the VIA, mapping data suggest that targeting immunodominant responses towards highly conserved regions of the HIV-1 proteome tended towards an increased ability to inhibit multiple clades of HIV-1 ex vivo.

## Conclusion

These data support the plausibility of inducing conserved CD8+ T cell responses using a consensus HIV-1 subtype A sequence in an adenovirus-based vector.

